# The gut microbiomes of Channel Island foxes and island spotted skunks exhibit fine‐scale differentiation across host species and island populations

**DOI:** 10.1002/ece3.11017

**Published:** 2024-02-14

**Authors:** Samantha Pasciullo Boychuck, Lara J. Brenner, Calypso N. Gagorik, Juliann T. Schamel, Stacy Baker, Elton Tran, Bridgett M. vonHoldt, Klaus‐Peter Koepfli, Jesús E. Maldonado, Alexandra L. DeCandia

**Affiliations:** ^1^ Biology, Georgetown University Washington DC USA; ^2^ The Nature Conservancy Ventura California USA; ^3^ Biology, Northern Arizona University Flagstaff Arizona USA; ^4^ National Park Service Ventura California USA; ^5^ Ecology and Evolutionary Biology Princeton University Princeton New Jersey USA; ^6^ Center for Species Survival Smithsonian's National Zoo & Conservation Biology Institute Front Royal Virginia USA; ^7^ Smithsonian‐Mason School of Conservation George Mason University Front Royal Virginia USA; ^8^ Center for Conservation Genomics Smithsonian's National Zoo & Conservation Biology Institute Washington DC USA

**Keywords:** coexistence, competition, host‐associated microbiome, mammal, microbial ecology, niche differentiation

## Abstract

California's Channel Islands are home to two endemic mammalian carnivores: island foxes (*Urocyon littoralis*) and island spotted skunks (*Spilogale gracilis amphiala*). Although it is rare for two insular terrestrial carnivores to coexist, these known competitors persist on both Santa Cruz Island and Santa Rosa Island. We hypothesized that examination of their gut microbial communities would provide insight into the factors that enable this coexistence, as microbial symbionts often reflect host evolutionary history and contemporary ecology. Using rectal swabs collected from island foxes and island spotted skunks sampled across both islands, we generated 16S rRNA amplicon sequencing data to characterize their gut microbiomes. While island foxes and island spotted skunks both harbored the core mammalian microbiome, host species explained the largest proportion of variation in the dataset. We further identified intraspecific variation between island populations, with greater differentiation observed between more specialist island spotted skunk populations compared to more generalist island fox populations. This pattern may reflect differences in resource utilization following fine‐scale niche differentiation. It may further reflect evolutionary differences regarding the timing of intraspecific separation. Considered together, this study contributes to the growing catalog of wildlife microbiome studies, with important implications for understanding how eco‐evolutionary processes enable the coexistence of terrestrial carnivores–and their microbiomes–in island environments.

## Graphical abstract

We examined the host‐associated microbiome of Channel Island foxes and island spotted skunks in the context of fine‐scale niche differentiation, as it is rare for two endemic, insular mesocarnivores to coexist. We found intraspecific variation between island populations, with greater differentiation observed between more specialist island spotted skunk populations compared to more generalist island fox populations. This pattern may reflect differences in resource utilization following fine‐scale niche differentiation, as well as evolutionary differences regarding the timing of intraspecific separation.

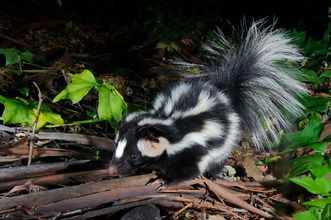

## 1 INTRODUCTION

In comparison to mainland habitats, islands often harbor significantly less vertebrate diversity (MacArthur & Wilson, 1967). This is likely due to the difficulty of island colonization, which can necessitate crossing large expanses of water or being introduced by humans (Lawlor, 1986). The prevalence of insular mammalian carnivorans (Order Carnivora) is especially low due to factors such as large body size, low carrying capacity, reduced vagility, and high extinction rate, with limited space and prey availability rendering niche differentiation difficult (Alcover & McMinn, 1994; Cardillo et al., 2008; Crooks & Van Vuren, 1995). This difficulty intensifies on small islands, as limited resources and heightened competition likely elevate carnivoran extinction rates further. Given their smaller size, mesocarnivores may be able to overcome some of these difficulties and act as apex predators on small islands (Roemer et al., 2009). However, theoretical expectations still suggest that coexistence between multiple carnivorans on the same island should be relatively rare.

**FIGURE 1:**
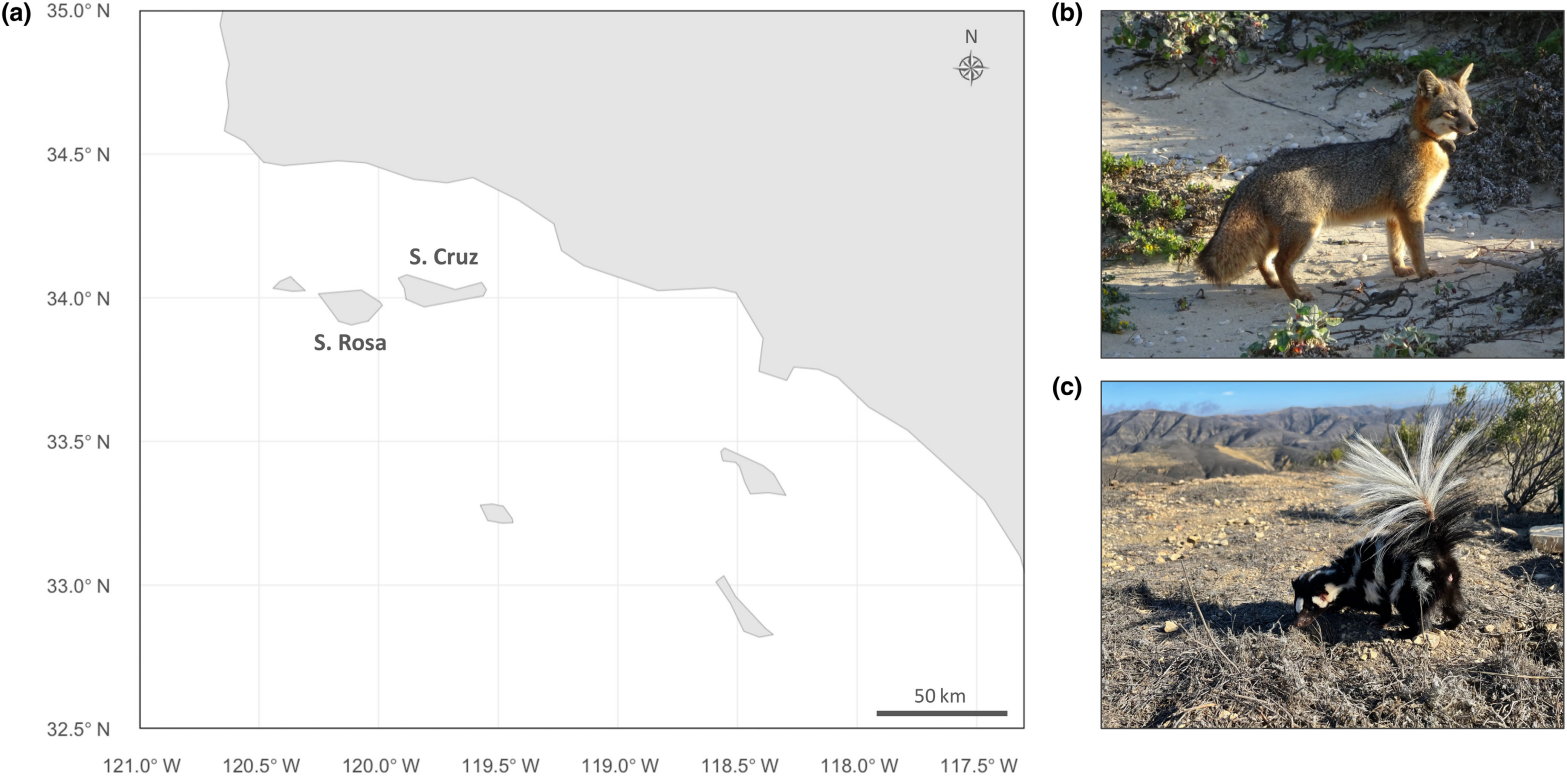
Channel Island foxes and island spotted skunks are endemic to Santa Rosa and Santa Cruz Islands. (a) A map of California's Channel Islands shows the proximity of Santa Rosa Island and Santa Cruz Island off the coast of southern California. This map was made using the *R* packages *rnaturalearth* (Massicotte & South, 2023), *ggplot2* (Wickham, 2016), and *ggspatial* (Dunnington, 2023). The top two terrestrial carnivores on these islands include (b) Channel Island foxes (*Urocyon littoralis*; image credit Stacy Baker) and (c) island spotted skunks (*Spilogale gracilis amphiala*; image credit Bridget Parrino).

Counter to these ecological patterns, Channel Island foxes (*Urocyon littoralis santacruzae* and *U. l. santarosae*) and island spotted skunks (*Spilogale gracilis amphiala*) coexist on two islands off the coast of southern California: Santa Cruz Island and Santa Rosa Island (Figure 1). Both mesocarnivore taxa are endemic to the Channel Islands, where they have coexisted alongside one another for at least 7000 years (Bolas, Quinn, et al., 2022; Floyd et al., 2011; Hofman et al., 2015). While island foxes are found on six of eight islands in the archipelago, island spotted skunks are only found on the two largest islands. Throughout their range, both species have undergone significant population fluctuations and currently exhibit low levels of genetic diversity, rendering them of particular conservation concern (Adams & Edmands, 2023; Floyd et al., 2011; Jones et al., 2013; Robinson et al., 2016). These species are also of interest to population ecologists, as they serve as a rare example of mammalian carnivore coexistence in island habitats.

This coexistence occurs amid known competition between island foxes and island spotted skunks. For example, direct competition was observed in the early 1990s following the colonization of two invasive species to the Channel Islands (Roemer et al., 2002). As populations of feral pigs increased, they served as abundant sources of prey that allowed golden eagles, an apex predator from the mainland, to colonize the archipelago. Golden eagles altered the trophic levels of the islands, turning the resident predators—namely, island foxes—into prey (Roemer et al., 2002). This intense predation on island foxes changed the competitive hierarchy between the island's endemic mesocarnivores by almost entirely removing island foxes from the ecosystem. In response, island spotted skunk numbers increased and they became more generalist in their diet and habitat use (Jones et al., 2008). The disruption of competitive dynamics between island foxes and island spotted skunks highlights the fragility of trophic structures within the Channel Islands. Additionally, the decrease in island fox populations and subsequent niche broadening of island spotted skunks demonstrates that foxes are likely the dominant competitor and that fine‐scale niche partitioning enables coexistence between these two terrestrial carnivores (Jones et al., 2008; Roemer et al., 2002).

Island foxes and island spotted skunks partition resources in numerous ways. While their niches overlap in habitat, temporal use, and diet, they maintain subtle, but important, distinctions. Regarding habitat, island foxes and island spotted skunks cohabitate within the coastal Mediterranean ecosystem on Santa Rosa and Santa Cruz Islands. Yet, island spotted skunks tend to be more specialized in their habitat use, while island foxes utilize all areas across the island (Crooks & Van Vuren, 1995). There may be further differences in microhabitat use, with island spotted skunks often found in areas with ground‐level cover in the form of rugged terrain or dense vegetation (Bolas, Sollmann, et al., 2022; Gagorik, 2020). Regarding temporal dynamics, island spotted skunks are largely nocturnal, whereas island foxes are cathemeral (active during both the day and night; Bolas, Sollmann, et al., 2022; Zhang et al., 2022), with differing degrees of seasonality. Finally, island spotted skunks are strictly carnivorous while island foxes are largely omnivorous (Crooks & Van Vuren, 1995; Cypher et al., 2014). Thus, island spotted skunks tend to be more specialist and island foxes tend to be more generalist along numerous ecological axes.

**FIGURE 2:**
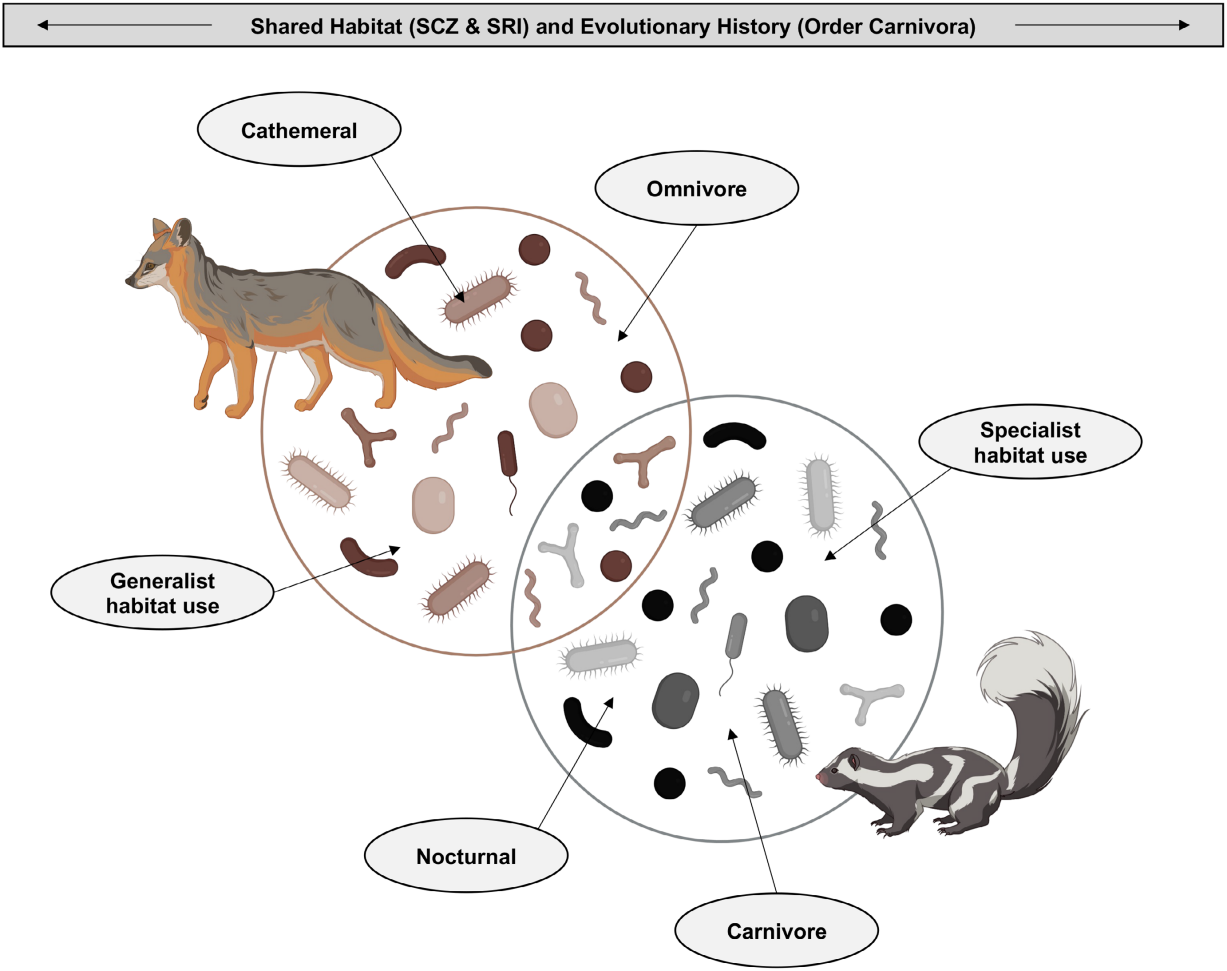
Fine‐scale niche differentiation between island foxes and island spotted skunks may influence their host‐associated microbiomes. Although they exhibit shared evolutionary history (both mesocarnivore species are within Order Carnivora) and inhabit the same two Channel Islands (SCZ = Santa Cruz Island; SRI = Santa Rosa Island), island foxes and island spotted skunks appear to coexist through niche partitioning. Island foxes tend to be more generalist in their habitat use, diet, and temporal activity, whereas island spotted skunks tend to be more specialist in these areas. Commensal microbial communities may reflect these differences, as depicted in this schematic created with BioRender.com.

Another way to explore niche differentiation between island foxes and island spotted skunks is to consider the composition of their host‐associated microbiomes. Microbiomes consist of all the internal and external microorganisms that have coevolved alongside their host (Berg et al., 2020). These microbes function in modulating metabolism (Mazidi et al., 2016), responding to disease (Williams et al., 2018), aiding in digestion (Hanning & Diaz‐Sanchez, 2015), mediating stress (Foster et al., 2017), and determining behavior (Archie & Tung, 2015). As such, characterizing the composition of host‐associated microbiomes can provide an array of information about the host species. It can reflect host diet (Kartzinel et al., 2019), health status (DeCandia et al., 2018, 2020; Lu et al., 2023), environmental context (Dallas & Warne, 2023), behavior (Moeller et al., 2023), and even evolutionary history (Brown et al., 2023; Figure 2). While specific taxa often differ between host species, the functional role of microbes is largely conserved among closely related hosts (Muegge et al., 2011). For example, carnivores require different bacteria than herbivores to assist in digestion, but both ultimately help in the breakdown of food (Zoelzer et al., 2021). Thus, the interplay between an organism's phylogenetic history and external environment manifest in the composition of their microbiome (Nishida & Ochman, 2018; Youngblut et al., 2019). Through understanding these host‐associated microbiomes, we can gain greater insights into the functioning of the whole organism, their evolutionary history, and how they fit within their broader ecological context.

In this study, we explored the role of gut microbial communities in niche differentiation between island foxes and island spotted skunks inhabiting Santa Cruz and Santa Rosa Islands. More specifically, we examined the relative contributions of evolutionary history (species) and environmental context (island) by comparing bacterial variation across host species and island populations using 16S rRNA amplicon sequencing data. We predicted that the microbiomes of these two host species would exhibit high‐level similarity due to their shared phylogenetic ancestry as members of the suborder Caniformia within the mammalian order Carnivora (Eizirik et al., 2010), low levels of genetic diversity, and overlapping habitats across two islands. However, we also anticipated finer scale specialization of microbes at lower taxonomic levels, following divergence of their ancestral lineages roughly 49 million years ago and more recent niche differentiation through subtle differences in diet, space use, and temporal activity patterns. Finally, we hypothesized that comparatively specialist island spotted skunks would exhibit lower microbial variation than more generalist island foxes. Considered together, this study provides important insights into the unusual coexistence between these two insular mammalian carnivores. This not only broadens our understanding of the eco‐evolutionary dynamics operating between hosts and their microbes in wild populations, but may further inform conservation monitoring and management of at‐risk species and the symbionts they harbor.

## 2 MATERIALS AND METHODS

### 2.1 Study area

The Channel Islands have a coastal Mediterranean climate with rainy winters and dry summers and are made up of rocky coasts, grasslands, shrublands, and woody forests (Junak et al., 2007). The islands have historically been subject to disruption, principally by humans, through the introduction of livestock and other invasive animal and plant species. This in turn has heavily impacted native flora and fauna communities on the islands. Santa Cruz Island (250 km^2^, the largest of the Channel Islands) and Santa Rosa Island (215 km^2^, the second largest) are part of the northern Channel Island archipelago and separated by a 9.6 km wide channel. The two islands were once connected as a super island called Santarosae that was separated from the mainland until sea level rise separated them from one another approximately 10,000 years ago after the end of the Pleistocene epoch (Erlandson et al., 2011; Reeder‐Myers et al., 2015). Santa Cruz Island (SCZ) is characterized by two east–west mountain ranges that bound a central valley, and hosts a variety of habitat types including grassland, chaparral, coastal sage scrub, oak woodland, and pine forests (Junak et al., 1995). Santa Rosa Island (SRI) is characterized by a single mountain chain running east–west through the middle of the island, with relatively flat marine terraces bisected by canyons and dominated by grassland on the north side, and more rugged terrain dominated by sagebrush scrub on the south side. Small pockets of native island oak and pine woodland are present. Both islands support a number of similar habitat types; however, Santa Rosa Island is less rugged and less topographically and vegetatively diverse compared to Santa Cruz Island. Grazing livestock were removed from most of Santa Cruz Island by the late 1980s, while grazers persisted on Santa Rosa Island until 2011. Thus, the majority of Santa Cruz Island is further advanced in vegetative recovery than Santa Rosa Island.

### 2.2 Sample collection

We sampled island foxes and island spotted skunks following live capture in Tomahawk box traps during annual island‐wide carnivore monitoring surveys conducted between June 2021 and February 2022. We inserted a Passive Integrated Transponder (PIT) tag in each individual animal for identification purposes and recorded basic demographic information including sex, weight, body condition, reproductive status, and approximate age for each animal. We used sterile BD BBL™ CultureSwab™ swabs (BD Biosciences, New Jersey, USA) to collect gut microbiome samples from the perianal area by gentling swabbing a total of 40 times per animal (i.e., 10 times before rotating the swab 90°). Perianal swabs serve as a reliable proxy for gut microbial communities, as described by Radhakrishnan et al. (2023) and previously used by DeCandia et al. (2020, 2021) and Lu et al. (2023). Upon field collection, we placed swabs on ice in a cooler bag to transfer samples to longer term frozen storage before DNA extraction. All field capture and handling efforts were conducted in accordance with island‐specific permissions and permits and following the guidelines established by the American Society of Mammalogists for the ethical use of wild mammals in research (Sikes et al., 2019). Samples on Santa Cruz Island were collected under a MOU with the California Department of Fish and Wildlife (CDFW) and Scientific Collecting Permit (No. 008981) issued by CDFW. Samples on Santa Rosa Island were collected under Channel Islands National Park Research Permit # CHIS‐2023‐SCI‐0017. All protocols were further approved by the Princeton University Institutional Animal Care and Use Committees (Princeton IACUC #3073).

### 2.3 DNA extraction, PCR, and sequencing

We extracted microbial DNA from perianal swabs using a modified Qiagen DNeasy PowerSoil HTP 96 Kit protocol (Qiagen Inc., Maryland, USA). We isolated swab tips using scissors sterilized by dunking blades in 80% ethanol solution before passing them through a flame. We randomized samples from different islands and species across PowerBead plates to minimize potential batch effects, and disrupted all samples and controls using a MiniBeadBeater‐96. We shook plates for 3 min, allowed 30 s of rest, rotated plates, and shook plates for an additional 3 min. We then followed the standard manufacturer protocol before eluting samples for 15 min at room temperature in 60 μL solution C6 preheated to 70°C.

We used polymerase chain reaction (PCR) to amplify and tag the 16S rRNA hypervariable 4 (V4) region with the barcoded forward (GTGCCAGCMGCCGCGGTAA) and reverse (TAATCTWTGGGVHCATCAGG) primers described in Caporaso et al. (2011). PCR reactions contained 5 μL HiFi HotStart ReadyMix (KAPA Biosystems, Massachusetts, USA), 3.2 μL primer mix (1.25 μM), and 2.0 μL template DNA. Extraction controls included sterile swab tips (negative controls) and DNA extracted from ZymoBIOMICS Microbial Community Standard (D6300; positive controls); and PCR controls included PCR reagents with no template DNA added (negative controls) and 1 μL of ZymoBIOMICS Microbial Community DNA Standard (D6305; positive controls; Zymo Research, California, USA). PCR cycling conditions included: initial denaturation of 94°C/3 min, touchdown cycling for 30 cycles of (94°C/45 s, 80–50°C/60 s, 72°C/90 s) decreasing 1°C each cycle, 12 cycles of (94°C/45 s, 50°C/60 s, 72°C/90 s), and a final extension of 72°C/10 min. To maximize yield and minimize PCR artifacts, each sample was run through PCR in duplicate and subsequently combined for quantification using a Qubit Flex Fluorometer (Invitrogen, Massachusetts, USA). We then pooled 40 ng of each barcoded library, used the QIAquick PCR Purification kit (Qiagen Inc., Maryland, USA) and E‐Gel 2% Size Selection Protocol (Invitrogen, Massachusetts, USA) to clean and size select libraries, and subsequently used Agencourt AMPure XP magnetic beads (Beckman Coulter, California, USA) to concentrate final libraries using a 1.25× bead to library ratio. Libraries were paired‐end sequenced (2×150 nt) across three sequencing plates on an Illumina MiSeq in the Princeton University Lewis Sigler Genomics Core Facility.

### 2.4 Sequence processing and analysis

We used a paired‐end, dual‐indexed barcode splitter implemented in *Galaxy* (Afgan et al., 2018) to demultiplex raw sequencing data, with allowance for one nucleotide mismatch between expected and observed barcodes. We then imported demultiplexed data into QIIME 2 v2021.11 (Bolyen et al., 2019; https://qiime2.org) and ran each sequencing plate through the *dada2 denoise‐paired* plugin (Callahan et al., 2016) to further process reads. This pipeline corrected likely sequencing errors, trimmed low quality bases, merged paired‐end reads, and removed chimeric reads. We then combined data from all three sequencing plates into one dataset for further filtering based on reads that appeared in positive and negative control samples.

We included six positive controls to assess possible sources of bias during DNA extraction (*n* = 3, ZymoBIOMICS Catalog No. D6300) and PCR amplification (*n* = 3, ZymoBIOMICS Catalog No. D6305). All six samples contained sequences from the three Gram‐negative and five Gram‐positive bacterial species contained within the mock community standards. This confirmed successful lysis, DNA extraction, amplification, and taxonomic identification of bacteria contained within our dataset. To identify potential contaminants, we implemented the R package *decontam* (Davis et al., 2018) using default parameters for the combined method that considers the frequency (distribution of the frequency of each sequence feature) and prevalence (presence/absence across samples) of potential contaminants, with sequencing plate used as a batch identifier. We subsequently filtered our dataset to remove the 32 amplicon sequence variants (ASVs) identified as possible contaminants by *decontam*; 130 ASVs with taxonomic labels of “mitochondria” or “chloroplasts”; any ASVs with a total frequency <10; and any ASV found in only one sample. We then removed all positive controls, negative controls, duplicate samples, and samples with fewer than 2500 reads. This produced a final dataset of 3,669,688 sequences and 1443 ASVs sequenced in 110 samples, including 21 foxes and 22 skunks sampled on Santa Cruz Island, and 33 foxes and 34 skunks sampled on Santa Rosa Island (Table S1).

### 2.5 Statistical analyses

We used QIIME 2 version 2022.8 (Bolyen et al., 2019; https://qiime2.org) to perform all downstream statistical analyses. We first implemented the *core‐metrics‐phylogenetic* function with sampling depth set to 2500 and a midpoint rooted phylogeny created with the *alignment* and *phylogeny* functions in order to calculate multiple measures of diversity. We set sampling depth to 2500 reads in order to retain all samples for downstream analysis (minimum read depth = 2505), and confirmed that rarefaction curves leveled off for the majority of samples prior to this depth (Figure S1). This indicates that our chosen sequencing depth adequately capture the bacterial diversity present in each sample. Alpha diversity metrics included observed features to measure richness (Hagerty et al., 2020), Pielou's evenness to measure species equitability (Pielou, 1966), and the Shannon Diversity Index as a combined measure of richness and evenness (Shannon, 1948). Beta diversity metrics included Bray–Curtis Dissimilarity to measure differences in microbial abundance (Bray & Curtis, 1957) and the Jaccard Index to measure differences in species presence (Jaccard, 1912). We calculated these five diversity measures in order to tease apart which aspects of diversity (e.g., bacterial presence vs. abundance) differed between our groups of interest. We performed multivariate significance testing using the *longitudinal anova* function (Bokulich, Dillon, et al., 2018) for alpha diversity measures and the *diversity adonis* function (multivariate PERMANOVA; Anderson, 2001; Oksanen et al., 2019) for beta diversity measures. These analyses contained the following variables: island, species, their interaction, sex, and sequencing plate. We visualized beta diversity differences using principal coordinate analysis implemented through the EMPeror plug‐in (Vázquez‐Baeza et al., 2013). All figures were created using the *qiime2R* (Bisanz, 2018) and *ggplot2* (Wickham, 2016) R packages.

We assigned taxonomy to representative ASVs by using a Naïve Bayes classifier trained on Greengenes 13_8 reference sequences (Bokulich, Kaehler, et al., 2018; DeSantis et al., 2006), as described previously (DeCandia et al., 2019, 2020, 2021; Lu et al., 2023). We then characterized the taxonomic composition of island fox and island spotted skunk gut microbiomes using the *taxa barplot* function and identified differentially abundant taxa within and between species using analysis of composition of microbiomes (ANCOM; Mandal et al., 2015) as implemented through the *ancom* function. ANCOM overcomes statistical hurdles associated with compositional datasets by calculating pairwise log‐ratios between all taxa present within sampling groups. It identifies which taxa consistently differ in abundance between groups by calculating the test statistic, *W*, as the number of times the null hypothesis of “no difference” is violated for a particular taxon. One of the underlying assumptions of ANCOM is that fewer than 25% of features (or ASVs) change between groups of interest. We confirmed that our dataset met this assumption for all four comparative analyses run. We calculated that 0.046% of features differed between island fox populations, 0.197% of features differed between island spotted skunk populations, and 0.359% and 0.386% of features differed between island foxes and island spotted skunks sampled on Santa Cruz Island and Santa Rosa Island, respectively. We then ran ANCOM at two taxonomic levels in order to obtain high‐level (class) and fine‐scale (genus) insights into which taxa significantly differ in abundance between groups of interest.

## 3 RESULTS

### 3.1 Island foxes exhibited higher alpha diversity than island spotted skunks across both Santa Cruz and Santa Rosa Islands

**FIGURE 3:**
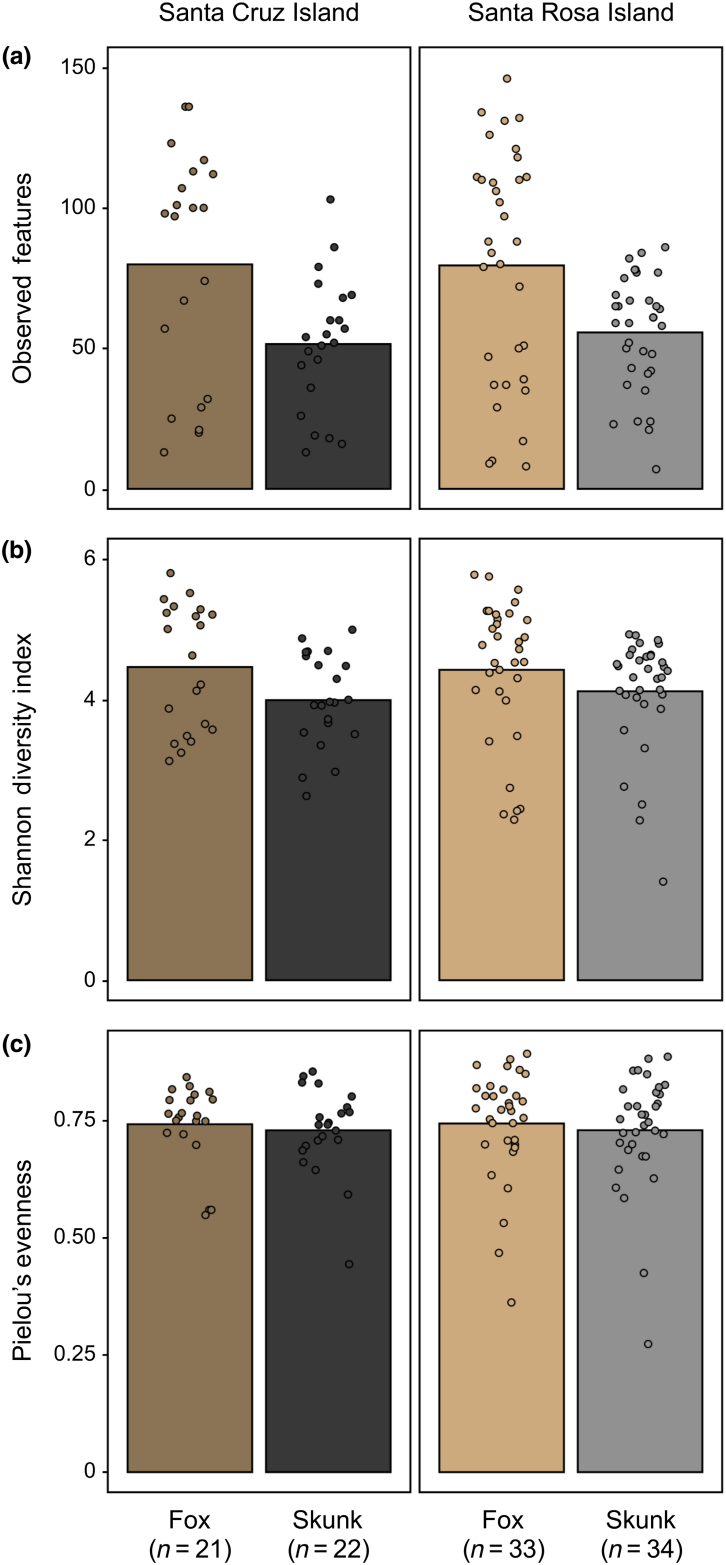
Alpha diversity differs by species, but not island. (a) Bacterial species richness (as measured by observed features) and (b) Shannon diversity significantly differed by species (ANOVA: richness, *F =* 44.448, df = 1, *p* < .001; Shannon diversity, *F =* 5.684, df = 1, *p =* .019), with foxes exhibiting higher diversity than skunks, but not by island (both *p* > .05). (c) Bacterial evenness differed by neither species nor island (both *p* > .05).

We found that bacterial richness and Shannon diversity significantly differed by species (ANOVA: richness, *F =* 44.448, df = 1, *p* < .001; Shannon diversity, *F =* 5.684, df = 1, *p =* .019) and sequencing plate (ANOVA: richness, *F =* 86.143, df = 2, *p* < .001; Shannon diversity, *F =* 7.468, df = 2, *p* < .001), with evenness only differing by sequencing plate (ANOVA: evenness, *F =* 16.476, df = 2, *p* < .001; Figure 3, Figure S2). In contrast, island, its interaction with species, and sex did not significantly predict alpha diversity across all three measures (all *p* > .05; Table S2). We further explored these patterns using pairwise Wilcoxon signed‐rank tests implemented between species and sequencing plates (Table S3). Between species, skunks exhibited significantly lower richness (pairwise Wilcoxon: *W* = 2093.0, adj.*p* = .002; Figure 3a) and Shannon diversity (pairwise Wilcoxon: *W* = 1969.0, adj.*p* = .014; Figure 3b). Between sequencing plates, plate CIF_S08 exhibited lower richness and Shannon diversity amid higher evenness compared to plates CIF_S09 and CIF_S10 (all adj.*p* < .05), which did not significantly differ from each other (all adj.*p* > .05; Figure S2, Table S3). This may be related to the average number of reads sequenced for each plate. Although data were rarefied prior to calculating diversity measures, average read depth after denoising was lower for samples sequenced on plate CIF_S08 (mean ± standard deviation: 17,065.1 ± 10,688.9) compared to plates CIF_S09 (50,559.5 ± 33,904.7) and CIF_S10 (35,937.0 ± 29,916.5).

### 3.2 Bacterial composition was significantly influenced by host species, with subtler interisland differences more pronounced in island spotted skunks compared to island foxes

**FIGURE 4:**
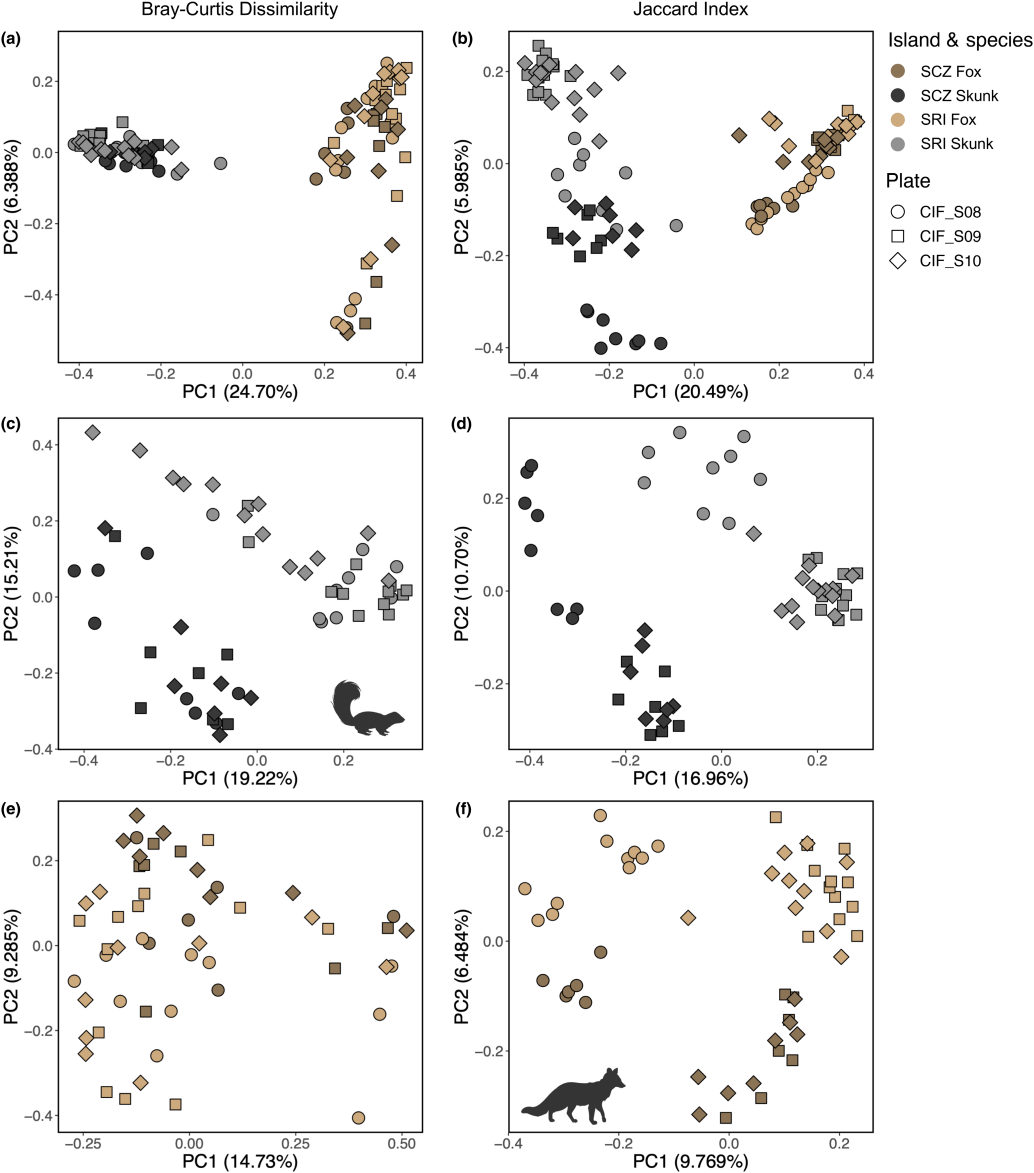
Beta diversity differs by species, island, and sequencing plate. We observed significant differences between species when comparing (a) bacterial abundance (as measured using Bray–Curtis dissimilarity; all *p* < .05) and (b) bacterial presence (as measured using the Jaccard index; all *p* < .05). Differences between island populations (SCZ = Santa Cruz Island; SRI = Santa Rosa Island) were greater within island spotted skunks (c, d) compared to island foxes (e, f), with additional clustering evident by sequencing plate (all *p* < .05). Fox and skunk silhouettes were created with BioRender.com.

Both Bray–Curtis dissimilarity and the Jaccard index were significantly predicted by species, island, their interaction, and sequencing plate, but not by sex (adonis: Table 1). Across both beta diversity measures, species explained the largest proportion of the variance in the dataset, with 24.0% for Bray–Curtis and 19.1% for Jaccard. Principal coordinate plots mirrored this result (Figure 4a,b), with separation by species clearly evident along PC1, which explained 24.7% of the variation for Bray–Curtis and 20.5% of the variation for Jaccard. Further examination of Bray–Curtis dissimilarity revealed separation between foxes by an unknown variable along PC2 (which explained 6.39% of the variation) and between skunks by island along PC3 (which explained 6.19% of the variation; Figure S3). Using Jaccard distances, we observed separation between skunks by island along PC2 (which explained 5.99% of the variation) and between sequencing plates along PC3 (which explained 4.18% of the variation; Figure S3).

**TABLE 1:** Results from multivariate beta diversity analyses for bacterial abundance (Bray–Curtis dissimilarity) and presence (Jaccard index) as implemented through the *diversity adonis* function in *QIIME2*.

	*F*	*R* ^2^	df	*p*
Bray–Curtis				
Island	6.418	.040	1	.001*
Species	38.404	.240	1	.001*
Island:Species	6.189	.039	1	.001*
Plate	2.473	.031	2	.004*
Sex	0.952	.006	1	.442
Residuals	–	.644	103	–
Jaccard				
Island	6.036	.040	1	.001*
Species	29.145	.191	1	.001*
Island:Species	5.779	.038	1	.001*
Plate	3.849	.050	2	.001*
Sex	1.112	.007	1	.246
Residuals	–	.674	103	–

*Note*: The *F‐*value (*F*), proportion of variation explained (*R* ^2^), degrees of freedom (df), and *p*‐value (*p*) are provided. Variables of interest included island, species, their interaction, sequencing plate, and sex.

* Statistical significance.

Given the strong influence of species on bacterial community structure, we created subsets of the data by species to look for finer scale intraspecific differentiation between island populations. Within island spotted skunks (Figure 4c,d), bacterial abundance and presence were significantly predicted by island and sequencing plate, but not sex (adonis: Table 2). The percent of the variation explained by island ranged from 14.6% to 15.9%, whereas sequencing plate ranged from 8.6% to 12.5%. We observed similar patterns in island foxes (Figure 4e,f), although island (5.6%–6.3%) and sequencing plate (6.7%–10.9%) explained lower proportions of variation (adonis: Table 2). Once again, we observed no significant influence of sex on either beta diversity measure (adonis: Table 2).

**TABLE 2:** Results from multivariate beta diversity analyses for bacterial abundance (Bray–Curtis dissimilarity) and presence (Jaccard index) as implemented through the *diversity adonis* function in *QIIME2* for species‐specific datasets.

	*F*	*R* ^2^	df	*p*
Island spotted skunks				
Bray–Curtis				
Island	10.945	.159	1	.001*
Plate	2.969	.086	2	.001*
Sex	1.043	.015	1	.359
Residuals	–	.740	51	–
Jaccard				
Island	10.464	.146	1	.001*
Plate	4.494	.125	2	.001*
Sex	1.391	.019	1	.094
Residuals	–	.710	51	–
Channel Island foxes				
Bray–Curtis				
Island	3.618	.063	1	.001*
Plate	1.931	.067	2	.002*
Sex	0.904	.016	1	.557
Residuals	–	.854	49	–
Jaccard				
Island	3.378	.056	1	.001*
Plate	3.253	.109	2	.001*
Sex	0.989	.017	1	.458
Residuals	–	.818	49	–

*Note*: The *F‐*value (*F*), proportion of variation explained (*R* ^*2*^), degrees of freedom (df), and *p*‐value (*p*) are provided. Variables of interest included island, sequencing plate, and sex.

* Statistical significance.

### 3.3 Island foxes and island spotted skunks exhibited the core mammalian microbiome, with the relative abundance of bacteria differing between host species and (for skunks) island populations

**FIGURE 5:**
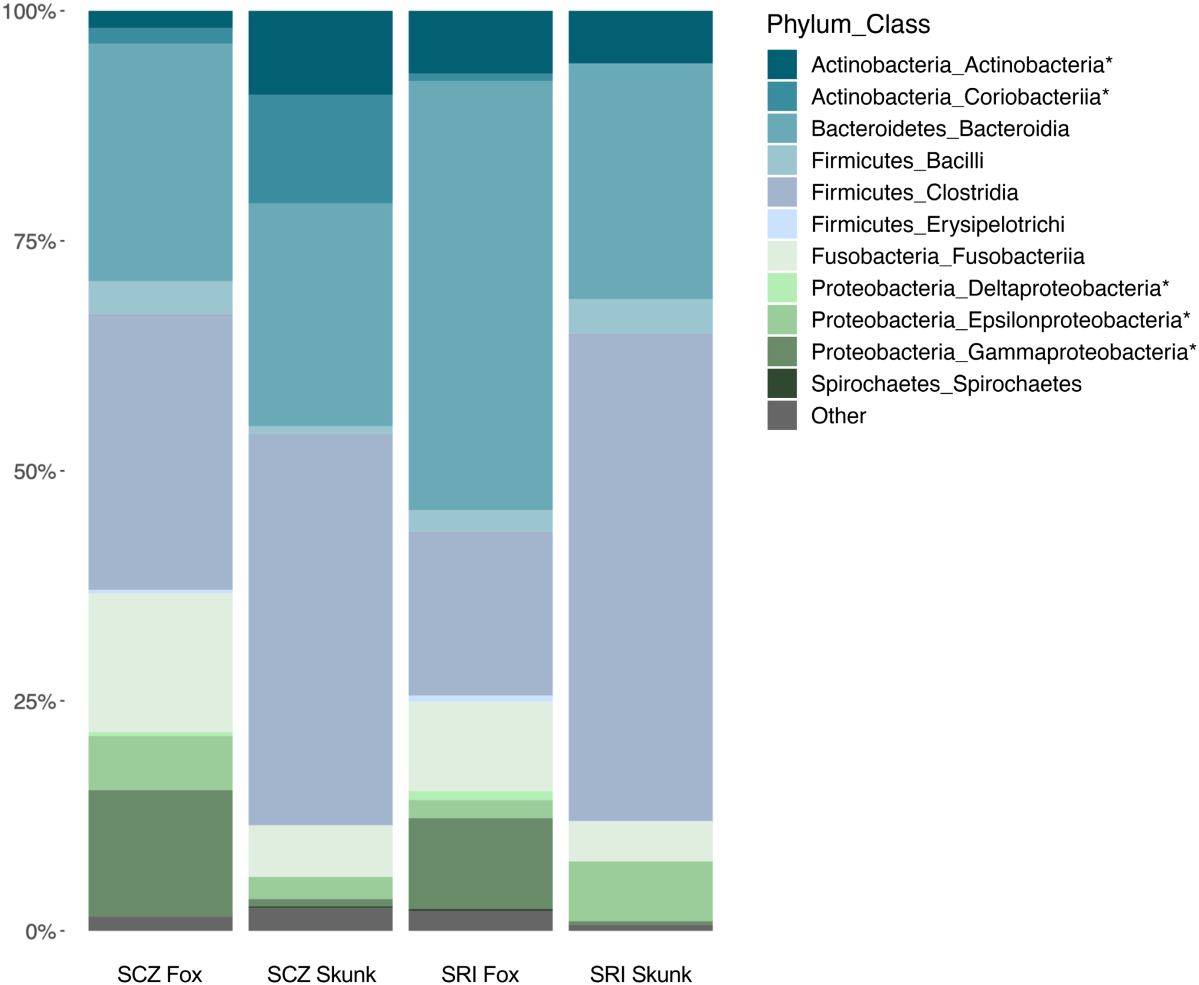
Island foxes and island spotted skunks exhibited the core mammalian microbiome, with differences in relative abundance observed between species and island populations. Each bar represents the combined samples collected from each island population studied, where SCZ indicates Santa Cruz Island and SRI indicates Santa Rosa Island. Each color denotes a distinct bacterial class, with all additional classes summed together and termed “Other.” Taxonomic groups annotated with an asterisk significantly differed between groups of interest during differential abundance testing with ANCOM.

Gut microbial communities of island foxes and island spotted skunks contained high proportions of Firmicutes (SCZ: Fox = 33.9%, Skunk = 43.4%; SRI: Fox = 20.8%, Skunk = 56.8%) and Bacteroidetes (SCZ: Fox = 26%, Skunk = 24.5%; SRI: Fox = 46.7%, Skunk = 25.6%). Dominant classes within these phyla included Bacilli, Clostridia, and (for foxes) Erysipelotrichi (Phylum: Firmicutes); and Bacteroidia (Phylum: Bacteroidetes; Figure 5). Additional phyla with relative abundances above 1% included Actinobacteria (SCZ: Fox = 3.6%, Skunk = 21%; SRI: Fox = 7.7%, Skunk = 5.8%), Proteobacteria (SCZ: Fox = 20.6%, Skunk = 4.1%; SRI: Fox = 13.3%, Skunk = 7.1%), and Fusobacteria (SCZ: Fox = 15.1%, Skunk = 5.6%; SRI: Fox = 9.7%, Skunk = 4.3%). Dominant classes within these phyla included Actinobacteria and Coriobacteriia (Phylum: Actinobacteria); Gammaproteobacteria, Epsilonproteobacteria, and Deltaproteobacteria (Phylum: Proteobacteria); and Fusobacteriia (Phylum: Fusobacteria; Figure 5). All additional phyla summed to less than 2% within each island population (SCZ: Fox = 0.8%, Skunk = 1.4%; SRI: Fox = 1.8%, Skunk = 0.4%).

While the presence of taxa was largely consistent across species and islands, their relative abundances often differed. We therefore used ANCOM to identify significantly differentially abundant taxa within each island and species at the class (Table 3) and genus (Table S4) levels. At the class level, within‐island comparisons between island foxes and island spotted skunks returned four unique classes. On Santa Cruz Island, island foxes harbored more Gammaproteobacteria (*W* = 54; 13.8% vs. 0.8%) and Deltaproteobacteria (*W* = 50; 0.4% vs. 0.009%) than island spotted skunks, while island spotted skunks harbored more Actinobacteria (*W* = 52; 9.2% vs. 1.9%) than island foxes. On Santa Rosa Island, island foxes again harbored more Gammaproteobacteria (*W* = 55; 9.9% vs. 0.4%) and Deltaproteobacteria (*W* = 57; 1% vs. 0.01%) than island spotted skunks, whereas island spotted skunks exhibited more Epsilonproteobacteria (*W* = 54; 6.5% vs. 2%). Similar analyses conducted within each species revealed island‐specific differences within island spotted skunks but not within island foxes (Table 3). Island spotted skunks exhibited a higher percentage of Coriobacteriia (*W* = 54; 11.8% vs. 0.08%) on Santa Cruz Island, with no classes significantly more abundant in Santa Rosa Island skunks.

**TABLE 3:** Five unique classes were significantly differentially abundant between groups of interest.

Phylum	Class	*W*	Dataset	Direction
Actinobacteria	Actinobacteria	52	SCZ only	Foxes < Skunks
Proteobacteria*	Deltaproteobacteria	50	SCZ only	Foxes > Skunks
Proteobacteria*	Gammaproteobacteria	54	SCZ only	Foxes > Skunks
Proteobacteria	Epsilonproteobacteria	54	SRI only	Foxes < Skunks
Proteobacteria*	Deltaproteobacteria	57	SRI only	Foxes > Skunks
Proteobacteria*	Gammaproteobacteria	55	SRI only	Foxes > Skunks
Actinobacteria	Coriobacteriia	54	Skunks only	SCZ > SRI
n/a	n/a	n/a	Foxes only	SCZ = SRI

*Note*: ANCOM was conducted at the class level within four subsets of data: Santa Cruz Island (SCZ) samples only, Santa Rosa Island (SRI) samples only, island spotted skunks only, and island foxes only. Across species comparisons returned four unique classes, with Deltaproteobacteria and Gammaproteobacteria consistently more abundant in foxes across both islands (as indicated by asterisks). Within species comparisons returned one differentially abundant class between skunk populations, with no significant results between island fox populations. The table includes the Phylum and Class of each significant result alongside the ANCOM test statistic (W, which indicates the number of times the null hypothesis was rejected for a given class), the dataset used for the analysis, and the direction of change (i.e., which group exhibited higher abundance).

ANCOM analyses at the genus level returned similar results. Interspecies comparisons yielded 22 and 48 differentially abundant genera when comparing island foxes to island spotted skunks sampled on Santa Cruz Island and Santa Rosa Island, respectively (Table S4). These included seven genera that were consistently more abundant in skunks and eight genera that were consistently more abundant in foxes across both islands, with additional taxa only identifiable at higher taxonomic levels. Comparative analyses conducted within each host species returned one significantly differentially abundant taxon each: *Collinsella* was more abundant in island spotted skunks sampled on Santa Cruz Island, and Fusobacteriia (genus unknown) was more abundant in island foxes sampled on Santa Cruz Island (Table S4).

## 4 DISCUSSION

This study is among the first to compare the gut microbiome of coexisting insular mesocarnivores within the context of fine‐scale niche differentiation. More specifically, we examined gut microbial diversity and composition between island foxes and island spotted skunks inhabiting Santa Cruz Island and Santa Rosa Island off the coast of southern California. We found that both island foxes and island spotted skunks harbor the core mammalian microbiome, while also exhibiting species‐specific microbial communities. Within species, island of origin significantly influenced gut microbial communities, with more pronounced differences observed in island spotted skunks compared to island foxes. This distinction may reflect the more specialized nature of island spotted skunk ecology, in contrast to the more generalist nature of island fox ecology. It may further reflect evolutionary processes operating during a longer period of separation between island spotted skunk populations, which diverged roughly 10,000 years ago, compared to island foxes, which colonized the islands roughly 7000 years ago (Bolas, Quinn, et al., 2022; Floyd et al., 2011; Hofman et al., 2015).

Regarding their similarities, the shared phylogenetic history of island foxes and island spotted skunks within the class Mammalia, order Carnivora, and suborder Caniformia (Eizirik et al., 2010) contributes to both host species harboring phyla that comprise the core mammalian microbiome. These phyla include Firmicutes, Actinobacteria, Bacteroidetes, and Proteobacteria, with the additional phylum Fusobacteria often reported in mammalian carnivores (Nelson et al., 2013). Interestingly, we observed higher levels of Fusobacteria in island foxes, which is consistent with other studies characterizing the microbiome of species within Canidae (Bragg et al., 2020; DeCandia et al., 2020, 2021). These results reflect the importance of evolutionary history in shaping the core features of mammalian microbiomes at higher taxonomic levels (Nishida & Ochman, 2018; Youngblut et al., 2019).

Yet amid these high‐level similarities, we also observed significant differentiation between island foxes and island spotted skunks. In fact, species identity explained the largest percentage of variation in our dataset by a significant margin, with 24% of microbial abundance and 19.1% of microbial presence attributed to host species. This result may reflect the evolutionary divergence between ancestral lineages of the Canidae and Mephitidae families roughly 49 million years ago, as well as ecological differences between these species that likely recur throughout their range. We posit that the reported patterns of diversity likely reflect both of these eco‐evolutionary processes, and in particular, the breadth of each species' ecological niche.

For example, examination of alpha diversity revealed a wider range of bacterial ASVs detected in island foxes compared to island spotted skunks, likely resulting from their more generalist resource utilization (Crooks & Van Vuren, 1995). Island foxes encounter a wider variety of food sources and environments that may contribute to their higher bacterial richness, as microbiomes are known to reflect an organism's diet and environmental setting (Kartzinel et al., 2019; Muegge et al., 2011). Conversely, as island spotted skunks are more specialized in resource use, they likely experience reduced exposure to these external sources of microbial variation, thus contributing to their lower bacterial richness. Their diet provides a likely candidate underlying this result. Compared to more omnivorous island foxes, island spotted skunk diets are mostly carnivorous and may therefore require fewer microbes to digest their smaller range of food sources (Crooks & Van Vuren, 1995). Previous research further showed that carnivore microbiomes tend to exhibit lower richness than omnivore microbiomes, as meat proteins are easier to digest than plant compounds (Nishida & Ochman, 2018; Youngblut et al., 2019). We therefore concluded that host species identity exerted a strong influence on gut microbial communities, likely due to their dietary differences.

These interspecific differences were further reflected in the relative abundances of bacterial taxa, as numerous microbes significantly differed between island foxes and island spotted skunks. At the taxonomic level of class, island foxes harbored higher relative abundances of Gammaproteobacteria and Deltaproteobacteria compared to their island spotted skunks counterparts on both Santa Cruz and Santa Rosa Islands. Gammaproteobacteria have previously been linked to coastal amphipod species (Mengoni et al., 2013) similar to those preyed upon by island foxes (Page et al., 2021). They have also been associated with soil invertebrates such as earthworms (Zhang et al., 2021), which were recently introduced to the Channel Islands (Paudel et al., 2016). These results potentially reflect a dietary preference toward these food sources among island foxes. It may also reflect evolutionary history, as Gammaproteobacteria have been isolated from other species within Canidae (Adams et al., 2021; DeCandia et al., 2021; Wetzels et al., 2021). While many of the Gammaproteobacteria results at the genus level lacked fine‐scale taxonomic resolution, the genus *Anaerobiospirillum* did appear in the Santa Rosa Island fox versus skunk comparison. This genus has been previously isolated from the gut microbiomes of harbor seal pups (*Phoca vitulina*; Pacheco‐Sandoval et al., 2022) and rural coyotes (*Canis latrans*; Sugden et al., 2020) with protein‐rich diets. These associations may again reflect a combination of evolutionary history and contemporary foraging ecology, as island fox diets likely contain food items sourced from marine and terrestrial habitats.

The other class consistently more abundant in island foxes, Deltaproteobacteria, has previously been linked to marine environments (Liu & Häggblom, 2018), and may further reflect island fox foraging behavior on beaches (Page et al., 2021). While genus level results only included Deltaproteobacteria in the Santa Rosa Island comparisons, we were able to obtain finer scale taxonomic resolution, as genera *Bilophila* and *Desulfovibrio* were both significantly more abundant in island foxes compared to island spotted skunks on this island. *Bilophila* has previously been associated with high‐fat diets in mice (Natividad et al., 2018), with an observed decrease in abundance after dietary supplementation with fish oil (Schots et al., 2020). In addition, *Desulfovibrio* has previously been isolated from marine sediments (Haouari et al., 2006; Thioye et al., 2017). Considered alongside the Gammaproteobacteria results reported above, this may again point to greater dietary breadth of island foxes foraging across terrestrial and marine environments.

We noted additional interspecific differences unique to each island. For example, island spotted skunks on Santa Rosa Island exhibited higher abundances of Epsilonproteobacteria. The genus largely driving this result was *Campylobacter*, which is often associated with enteritis in humans that come into contact with soil, untreated water sources, or animals (Butzler, 2004). In island spotted skunks, this may derive from their local environment or prey items, as *Campylobacter* has previously been isolated from wild mice (Song et al., 2021). On Santa Cruz Island, Actinobacteria proved to be more abundant in island spotted skunks when performing analyses at the class level. However, genus‐level analyses consistently returned two genera within Actinobacteria (*Arcanobacterium* and *Mobiluncus*) as significantly more abundant in island spotted skunks compared to island foxes across both islands. As Actinobacteria is one of the most abundant bacterial taxa in soils (Lewin et al., 2016), this may reflect habitat use by island spotted skunks, which spend more time in ravines or covered places while foraging for soil‐dwelling prey (Bolas, Sollmann, et al., 2022; Crooks & Van Vuren, 1995). We therefore posit that the increased presence of certain bacterial classes in island foxes and island spotted skunks may derive from eco‐evolutionary differences in their population histories, diets, and preferred habitat types, as well.

While species identity was the strongest predictor of bacterial composition, we also observed intraspecific differences between island populations. This pattern was more pronounced in island spotted skunks, where 15.9% of bacterial abundance and 14.6% of species presence were attributed to island of origin, compared to 6.3% of bacterial abundance and 5.6% of species presence in island foxes. We again attribute this near threefold difference to two likely factors: The longer separation time between island populations and the more specialist ecology of island spotted skunks, as subtle differences between island habitats may have a stronger effect on their gut microbial communities. This contrasts with the more recent colonization events and more generalist ecology of island foxes, which harbor a greater diversity of microbes that may reflect their interactions with numerous dietary items and microhabitats across both islands. This likely has a homogenizing effect on their gut microbial communities, although we do still find evidence of intraspecific variation, as previously reported in island fox gut microbial communities (Adams et al., 2021).

Differential abundance testing supported these overarching patterns. At the class level, intraspecific comparisons revealed one bacterial class significantly differing between island spotted skunk populations amid no significant differences between island fox populations. At the genus level, each analyses returned one significant result for each intraspecific comparison. Within skunks, the Santa Cruz Island population harbored significantly more Coriobacteriia, which has been linked with the addition of crickets to the diet of domestic dogs as it helps break down chitin in insect exoskeletons (Jarett et al., 2019). These results suggest that Santa Cruz Island spotted skunks may be eating larger proportions of native arthropod species than their Santa Rosa counterparts. This link between Coriobacteriia and crickets is particularly compelling, as previous research reported Jerusalem crickets (*Stenopelmatus fuscus*) as the most frequently consumed arthropod by Santa Cruz Island spotted skunks (Crooks & Van Vuren, 1995). Genus‐level analyses supported this result, as *Collinsella* (class: Coriobacteriia) was more abundant in Santa Cruz Island foxes. This genus has been previously associated with insect‐rich diets across host‐taxa, including poultry experimentally fed insect larvae (Colombino et al., 2021) and dogs experimentally fed crickets, as cited above (Jarett et al., 2019). Within foxes, genus‐level analyses found that one taxon (Fusobacteriaceae, genus unknown) was significantly more abundant in Santa Cruz Island foxes compared to Santa Rosa Island foxes. The biological significance of this result remains uncertain, as this family is commonly associated with canid gut microbiomes across host taxa (Bragg et al., 2020; DeCandia et al., 2020, 2021; Nelson et al., 2013). Considered alongside interspecific results, these findings from intraspecific comparisons further underscore the importance of evolutionary history and dietary preference as a driver of gut microbial composition within and between host populations and species.

The final factor significantly influencing microbial diversity in our dataset was sequencing plate, which represents a statistical relict of batch effects. Throughout DNA extraction, library preparation, and sequencing, we took numerous measures to minimize batch effects. These included standardization of all laboratory protocols, randomization of samples collected from different species and island populations across plates, running PCRs in duplicate and combining the resulting product, pooling and cleaning final libraries on the same day, and sequencing all libraries on the same Illumina MiSeq machine. We additionally performed multiple data processing steps (e.g., denoising raw reads, removing potential contaminants, and rarefying sequences prior to calculating diversity measures) to clean and standardize our dataset. However, we still observed significantly lower diversity in samples sequenced on sequencing plate CIF_S08 compared to the other two plates (i.e., CIF_S09 and CIF_S10). While this batch effect did not preclude us from discovering meaningful biological insights about the microbial ecology of island foxes and island spotted skunks, it does highlight the risk of batch effects in amplicon sequencing datasets, as noted by other authors (Regueira‐Iglesias et al., 2023; Wang & LêCao, 2020).

In summary, this study characterized the gut microbiomes of island foxes and island spotted skunks across two of California's Channel Islands: Santa Cruz Island and Santa Rosa Island. We identified the core mammalian microbiome present in both species likely due to their shared evolutionary history, and reported evidence of niche differentiation likely due to differences in diet and habitat use between these coexisting mesocarnivores. We found that host species was the primary driver of microbial differentiation within our dataset, and additionally reported intraspecific variation between island populations. These intraspecific differences were more pronounced in the longer separated and more specialist island spotted skunks compared to the more recently colonized and more generalist island foxes. These results therefore support the claim that host evolutionary history and contemporary ecology (i.e., diet, habitat use, and local environmental context) all play a role in shaping the gut microbiome. Commensal bacteria likely reflect—and perhaps even contribute to—the fine‐scale niche differentiation that allows these insular terrestrial carnivores to coexist. To better understand this ecological rarity, we recommend further study of the diet, behavior, and bacteria colonizing these known competitors, with careful attention to minimizing batch effects. This will shed additional light on the eco‐evolutionary processes that have enabled their coexistence since initial colonization millennia ago, and will hopefully facilitate future coexistence for many millennia to come.

## AUTHOR CONTRIBUTIONS

**Samantha Pasciullo Boychuck:** Formal analysis (equal); writing – original draft (lead); writing – review and editing (equal). **Lara J. Brenner:** Conceptualization (equal); data curation (equal); writing – original draft (equal); writing – review and editing (equal). **Calypso N. Gagorik:** Conceptualization (equal); data curation (equal); writing – original draft (equal); writing – review and editing (equal). **Juliann T. Schamel:** Conceptualization (equal); data curation (equal); writing – original draft (equal); writing – review and editing (equal). **Stacy Baker:** Conceptualization (equal); data curation (equal); writing – original draft (equal); writing – review and editing (equal). **Elton Tran:** Formal analysis (equal). **Bridgett M. vonHoldt:** Conceptualization (equal); supervision (equal); writing – review and editing (equal). **Klaus‐Peter Koepfli:** Conceptualization (equal); supervision (equal); writing – review and editing (equal). **Jesús E. Maldonado:** Conceptualization (equal); supervision (equal); writing – review and editing (equal). **Alexandra L. DeCandia:** Conceptualization (equal); data curation (equal); formal analysis (lead); funding acquisition (lead); project administration (lead); supervision (equal); visualization (lead); writing – original draft (lead); writing – review and editing (equal).

## CONFLICT OF INTEREST STATEMENT

The authors have no known conflicts of interest.

## BENEFITS GENERATED

Benefits from this research accrue from the sharing of our data and results on public databases as described above.

## Supporting information


Appendix S1

## Data Availability

Sequencing data analyzed in this study is publicly available through the NCBI Sequence Read Archive (SRA) under BioProject PRJNA1012705. Demultiplexed forward and reverse reads are available for each sample (BioSamples SAMN37271150 through SAMN37271259), with SRA accession numbers SRR25897939 through SRR25898048. Metadata are also stored in the SRA (BioProject PRJNA1012705) using the MIMARKS survey host‐associated 6.0 package.
