# Supplementary material for: The gut microbiomes of Channel Island foxes and island spotted skunks exhibit fine‐scale differentiation across host species and island populations
Source: Ecol Evol. 2024 Feb 14;14(2):e11017. doi: 10.1002/ece3.11017 (PMC10867392; doi:10.1002/ece3.11017)
Supplement: Supplementary file 1 — Appendix S1 [file ECE3-14-e11017-s001.docx]

**Supplemental Tables and Figures**

**Table S1. Metadata variables for the 110 samples included in this study.** Sample identifier, animal identifier, island (where SCZ = Santa Cruz Island and SRI = Santa Rosa Island), species, sequencing plate, and sex are provided.

| **Sample ID** | **Animal ID** | **Island** | **Species** | **Plate** | **Sex** |
| --- | --- | --- | --- | --- | --- |
| ALD1326 | Fox_D2535 | SCZ | Fox | CIF_S8 | F |
| ALD1329 | Fox_65297 | SCZ | Fox | CIF_S8 | M |
| ALD1459 | Fox_59433 | SCZ | Fox | CIF_S8 | M |
| ALD1462 | Fox_65301 | SCZ | Fox | CIF_S8 | M |
| ALD1537 | Fox_59419 | SCZ | Fox | CIF_S8 | M |
| ALD1540 | Fox_30402 | SCZ | Fox | CIF_S8 | F |
| ALD1327 | Fox_30290 | SCZ | Fox | CIF_S9 | F |
| ALD1460 | Fox_65259 | SCZ | Fox | CIF_S9 | M |
| ALD1463 | Fox_C6B71 | SCZ | Fox | CIF_S9 | F |
| ALD1532 | Fox_82B76 | SCZ | Fox | CIF_S9 | M |
| ALD1535 | Fox_59430 | SCZ | Fox | CIF_S9 | F |
| ALD1538 | Fox_59462 | SCZ | Fox | CIF_S9 | M |
| ALD1541 | Fox_D060D | SCZ | Fox | CIF_S9 | M |
| ALD1328 | Fox_59434 | SCZ | Fox | CIF_S10 | F |
| ALD1458 | Fox_59609 | SCZ | Fox | CIF_S10 | F |
| ALD1461 | Fox_59476 | SCZ | Fox | CIF_S10 | F |
| ALD1464 | Fox_27055 | SCZ | Fox | CIF_S10 | M |
| ALD1533 | Fox_91204 | SCZ | Fox | CIF_S10 | M |
| ALD1536 | Fox_65291 | SCZ | Fox | CIF_S10 | F |
| ALD1539 | Fox_59400 | SCZ | Fox | CIF_S10 | UNK |
| ALD1542 | Fox_50D5F | SCZ | Fox | CIF_S10 | M |
| ALD1302 | Skunk_65280 | SCZ | Skunk | CIF_S8 | M |
| ALD1307 | Skunk_65257 | SCZ | Skunk | CIF_S8 | M |
| ALD1332 | Skunk_65306 | SCZ | Skunk | CIF_S8 | M |
| ALD1335 | Skunk_65334 | SCZ | Skunk | CIF_S8 | F |
| ALD1417 | Skunk_65330 | SCZ | Skunk | CIF_S8 | M |
| ALD1420 | Skunk_65312 | SCZ | Skunk | CIF_S8 | F |
| ALD1423 | Skunk_65318 | SCZ | Skunk | CIF_S8 | M |
| ALD1543 | Skunk_65328 | SCZ | Skunk | CIF_S8 | F |
| ALD1297 | Skunk_65309 | SCZ | Skunk | CIF_S9 | M |
| ALD1300 | Skunk_65349 | SCZ | Skunk | CIF_S9 | M |
| ALD1341 | Skunk_65325 | SCZ | Skunk | CIF_S9 | F |
| ALD1344 | Skunk_65294 | SCZ | Skunk | CIF_S9 | M |
| ALD1418 | Skunk_65274 | SCZ | Skunk | CIF_S9 | F |
| ALD1421 | Skunk_65288 | SCZ | Skunk | CIF_S9 | M |
| ALD1424 | Skunk_65303 | SCZ | Skunk | CIF_S9 | M |
| ALD1334 | Skunk_65347 | SCZ | Skunk | CIF_S10 | M |
| ALD1337 | Skunk_65333 | SCZ | Skunk | CIF_S10 | M |
| ALD1342 | Skunk_65265 | SCZ | Skunk | CIF_S10 | M |
| ALD1416 | Skunk_65302 | SCZ | Skunk | CIF_S10 | F |
| ALD1419 | Skunk_65262 | SCZ | Skunk | CIF_S10 | M |
| ALD1422 | Skunk_65337 | SCZ | Skunk | CIF_S10 | M |
| ALD1425 | Skunk_65279 | SCZ | Skunk | CIF_S10 | F |
| ALD1308 | Fox_99232 | SRI | Fox | CIF_S8 | F |
| ALD1495 | Fox_78746 | SRI | Fox | CIF_S8 | M |
| ALD1498 | Fox_16329 | SRI | Fox | CIF_S8 | F |
| ALD1501 | Fox_20976 | SRI | Fox | CIF_S8 | M |
| ALD1505 | Fox_24121 | SRI | Fox | CIF_S8 | M |
| ALD1511 | Fox_44190 | SRI | Fox | CIF_S8 | F |
| ALD1514 | Fox_24375 | SRI | Fox | CIF_S8 | M |
| ALD1517 | Fox_16520 | SRI | Fox | CIF_S8 | M |
| ALD1520 | Fox_21266 | SRI | Fox | CIF_S8 | M |
| ALD1523 | Fox_80161 | SRI | Fox | CIF_S8 | M |
| ALD1526 | Fox_30734 | SRI | Fox | CIF_S8 | M |
| ALD1311 | Fox_59907 | SRI | Fox | CIF_S9 | M |
| ALD1496 | Fox_17857 | SRI | Fox | CIF_S9 | F |
| ALD1499 | Fox_62159 | SRI | Fox | CIF_S9 | F |
| ALD1503 | Fox_20472 | SRI | Fox | CIF_S9 | M |
| ALD1506 | Fox_24407 | SRI | Fox | CIF_S9 | F |
| ALD1509 | Fox_79820 | SRI | Fox | CIF_S9 | M |
| ALD1512 | Fox_18181 | SRI | Fox | CIF_S9 | M |
| ALD1515 | Fox_21310 | SRI | Fox | CIF_S9 | M |
| ALD1518 | Fox_99896 | SRI | Fox | CIF_S9 | M |
| ALD1521 | Fox_21050 | SRI | Fox | CIF_S9 | M |
| ALD1524 | Fox_21384 | SRI | Fox | CIF_S9 | M |
| ALD1527 | Fox_06182 | SRI | Fox | CIF_S9 | F |
| ALD1312 | Fox_20999 | SRI | Fox | CIF_S10 | M |
| ALD1497 | Fox_80348 | SRI | Fox | CIF_S10 | M |
| ALD1504 | Fox_60571 | SRI | Fox | CIF_S10 | M |
| ALD1510 | Fox_24085 | SRI | Fox | CIF_S10 | F |
| ALD1513 | Fox_79503 | SRI | Fox | CIF_S10 | M |
| ALD1516 | Fox_59989 | SRI | Fox | CIF_S10 | M |
| ALD1519 | Fox_18726 | SRI | Fox | CIF_S10 | M |
| ALD1522 | Fox_20894 | SRI | Fox | CIF_S10 | M |
| ALD1525 | Fox_42866 | SRI | Fox | CIF_S10 | M |
| ALD1528 | Fox_80322 | SRI | Fox | CIF_S10 | M |
| ALD1429 | Skunk_21402 | SRI | Skunk | CIF_S8 | M |
| ALD1432 | Skunk_20904 | SRI | Skunk | CIF_S8 | F |
| ALD1466 | Skunk_18491 | SRI | Skunk | CIF_S8 | M |
| ALD1469 | Skunk_79852 | SRI | Skunk | CIF_S8 | M |
| ALD1472 | Skunk_18349 | SRI | Skunk | CIF_S8 | M |
| ALD1475 | Skunk_18107 | SRI | Skunk | CIF_S8 | M |
| ALD1478 | Skunk_18424 | SRI | Skunk | CIF_S8 | M |
| ALD1485 | Skunk_21298 | SRI | Skunk | CIF_S8 | M |
| ALD1491 | Skunk_21618 | SRI | Skunk | CIF_S8 | M |
| ALD1309 | Skunk_79983 | SRI | Skunk | CIF_S9 | F |
| ALD1427 | Skunk_20951 | SRI | Skunk | CIF_S9 | M |
| ALD1430 | Skunk_21331 | SRI | Skunk | CIF_S9 | M |
| ALD1467 | Skunk_52236 | SRI | Skunk | CIF_S9 | M |
| ALD1470 | Skunk_21197 | SRI | Skunk | CIF_S9 | M |
| ALD1473 | Skunk_21208 | SRI | Skunk | CIF_S9 | M |
| ALD1476 | Skunk_21557 | SRI | Skunk | CIF_S9 | M |
| ALD1480 | Skunk_39724 | SRI | Skunk | CIF_S9 | M |
| ALD1483 | Skunk_21630 | SRI | Skunk | CIF_S9 | M |
| ALD1486 | Skunk_21235 | SRI | Skunk | CIF_S9 | F |
| ALD1489 | Skunk_21624 | SRI | Skunk | CIF_S9 | M |
| ALD1492 | Skunk_21451 | SRI | Skunk | CIF_S9 | M |
| ALD1310 | Skunk_21528 | SRI | Skunk | CIF_S10 | F |
| ALD1428 | Skunk_39211 | SRI | Skunk | CIF_S10 | F |
| ALD1431 | Skunk_52288 | SRI | Skunk | CIF_S10 | M |
| ALD1465 | Skunk_21434 | SRI | Skunk | CIF_S10 | M |
| ALD1468 | Skunk_60199 | SRI | Skunk | CIF_S10 | M |
| ALD1471 | Skunk_20956 | SRI | Skunk | CIF_S10 | F |
| ALD1474 | Skunk_18147 | SRI | Skunk | CIF_S10 | M |
| ALD1477 | Skunk_39191 | SRI | Skunk | CIF_S10 | M |
| ALD1481 | Skunk_21217 | SRI | Skunk | CIF_S10 | M |
| ALD1484 | Skunk_60054 | SRI | Skunk | CIF_S10 | F |
| ALD1487 | Skunk_21803 | SRI | Skunk | CIF_S10 | M |
| ALD1490 | Skunk_21079 | SRI | Skunk | CIF_S10 | M |
| ALD1493 | Skunk_21214 | SRI | Skunk | CIF_S10 | F |

**Figure S1. Rarefaction curves confirm that sampling depth adequately captures diversity.** Rarefaction analyses using (A) observed features as a measure of richness, (B) the Shannon diversity index as a measure of richness and equitability, and (C) Pielou’s evenness as a measure of equitability show that the sampling depth of 2500 provides adequate coverage for capturing bacterial diversity in Channel Island fox and island spotted skunk gut microbial communities.

**Figure S2. Alpha diversity differs across sequencing plates.** (A) Bacterial species richness (as measured by observed features), (B) Shannon diversity, and (C) Pielou’s Evenness significantly differed by sequencing plate (ANOVA: richness, *F=*86.143, *df*=2, *p*<0.001; Shannon diversity, *F=*7.468, *df*=2, *p*<0.001; evenness, *F=*16.476, *df*=2, *p*<0.001). This result was driven by lower richness and Shannon diversity – but higher evenness – in sequencing plate CIF_S08.

**Table S2. Results from multivariate alpha diversity analyses** for bacterial richness, Shannon diversity, and Pielou’s evenness implemented through the *longitudinal anova* function in *QIIME2*. Sum of squares (Sum Sq.), degrees of freedom (*df*), *F-*value (*F*), and *p*-value (*p*) are provided. Variables of interest include island, species, their interaction, sequencing plate, and sex. Asterisks indicate statistical significance.

|  |  | **Sum Sq.** | ***df*** | ***F*** | ***p*** |
| --- | --- | --- | --- | --- | --- |
| **Richness** | **Island** | 6.340 | 1 | 0.015 | 0.902 |
|  | **Species** | 18651.232 | 1 | 44.448 | <0.001* |
|  | **Island:Species** | 77.592 | 1 | 0.185 | 0.668 |
|  | **Plate** | 72295.106 | 2 | 86.143 | <0.001* |
|  | **Sex** | 41.298 | 1 | 0.098 | 0.754 |
|  | **Residual** | 43220.886 | 103 |  |  |
|  |  |  |  |  |  |
| **Shannon** | **Island** | 0.018 | 1 | 0.026 | 0.872 |
|  | **Species** | 3.848 | 1 | 5.684 | 0.019* |
|  | **Island:Species** | 0.041 | 1 | 0.060 | 0.807 |
|  | **Plate** | 10.112 | 2 | 7.468 | <0.001* |
|  | **Sex** | 0.003 | 1 | 0.005 | 0.945 |
|  | **Residual** | 69.738 | 103 |  |  |
|  |  |  |  |  |  |
| **Evenness** | **Island** | <0.001 | 1 | 0.027 | 0.869 |
|  | **Species** | 0.004 | 1 | 0.456 | 0.501 |
|  | **Island:Species** | 0.002 | 1 | 0.195 | 0.660 |
|  | **Plate** | 0.310 | 2 | 16.476 | <0.001* |
|  | **Sex** | <0.001 | 1 | 0.044 | 0.835 |
|  | **Residual** | 0.969 | 103 |  |  |

**Table S3. Results from pairwise Wilcoxon Signed-Rank tests** for bacterial richness, Shannon diversity, and Pielou’s evenness. The test statistic (*W*), *p-*value (*p*), and adjusted *p*-value (*p-adj)* following correction for multiple testing (Benjamini-Hochberg false discover rate) are provided. Abbreviations SCZ = Santa Cruz Island and SRI = Santa Rosa Island. The three sequencing plates included in these analyses include CIF_S08, CIF_S09, and CIF_S10. Asterisks indicate statistical significance.

|  |  | ***W*** | ***p*** | ***p-adj*** |
| --- | --- | --- | --- | --- |
| **Richness** | **SRI-SCR** | 1383.5 | 0.729 | 0.820 |
|  | **Skunk-Fox** | 2093.0 | <0.001 | 0.002* |
|  | **Male-Female** | 1362.5 | 0.453 | 0.668 |
|  | **CIF_S09-CIF_S08** | 9.0 | <0.001 | <0.001* |
|  | **CIF_S10-CIF_S08** | 41.5 | <0.001 | <0.001* |
|  | **CIF_S10-CIF_S09** | 784.0 | 0.523 | 0.668 |
|  |  |  |  |  |
| **Shannon** | **SRI-SCR** | 1318.0 | 0.455 | 0.668 |
|  | **Skunk-Fox** | 1969.0 | 0.006 | 0.014* |
|  | **Male-Female** | 1289.0 | 0.790 | 0.836 |
|  | **CIF_S09-CIF_S08** | 323.0 | <0.001 | <0.001* |
|  | **CIF_S10-CIF_S08** | 379.0 | 0.002 | 0.006* |
|  | **CIF_S10-CIF_S09** | 782.0 | 0.539 | 0.668 |
|  |  |  |  |  |
| **Evenness** | **SRI-SCR** | 1344.0 | 0.557 | 0.668 |
|  | **Skunk-Fox** | 1678.0 | 0.322 | 0.645 |
|  | **Male-Female** | 1239.0 | 0.955 | 0.955 |
|  | **CIF_S09-CIF_S08** | 1116.0 | <0.001 | <0.001* |
|  | **CIF_S10-CIF_S08** | 1151.0 | <0.001 | <0.001* |
|  | **CIF_S10-CIF_S09** | 799.0 | 0.429 | 0.668 |

**Figure S3. Beta diversity differs by species, island, and sequencing plate.** We observed significant differences between species, island (where SCZ = Santa Cruz Island and SRI = Santa Rosa Island), and sequencing plate when comparing (A) bacterial abundance (as measured using Bray-Curtis Dissimilarity; all *p*<0.05) and (B) bacterial presence (as measured using the Jaccard Index; all *p*<0.05).

**Table S4. Numerous genera were significantly differentially abundant between groups of interest.** ANCOM was conducted at the genus level within four subsets of data: Santa Cruz Island (SCZ) samples only, Santa Rosa Island (SRI) samples only, island spotted skunks only, and island foxes only. Across species comparisons returned 22 (SCZ only) and 48 (SRI only) unique genera, with numerous taxa consistently more abundant in one species or the other (as indicated by asterisks). Within species comparisons returned one differentially abundant genus between skunk populations (*Collinsella*) and between fox populations (Fusobacteriia; genus unknown). The table includes the Phylum, Class, and Genus of each significant result alongside the ANCOM test statistic (W, which indicates the number of times the null hypothesis was rejected for a given class), the dataset used for the analysis, and the direction of change (*i.e.*, which group exhibited higher abundance).

|  | **Phylum** | **Class** | **Genus** | **W** | **Dataset** | **Direction** |
| --- | --- | --- | --- | --- | --- | --- |
| * | Actinobacteria | Actinobacteria | *Arcanobacterium* | 280 | SCZ only | Foxes < Skunks |
| * | Actinobacteria | Actinobacteria | *Mobiluncus* | 281 | SCZ only | Foxes < Skunks |
|  | Bacteroidetes | Bacteroidia | *-* | 272 | SCZ only | Foxes < Skunks |
| * | Bacteroidetes | Bacteroidia | *Prevotella* | 255 | SCZ only | Foxes < Skunks |
|  | Firmicutes | Clostridia | *-* | 281 | SCZ only | Foxes < Skunks |
| * | Firmicutes | Clostridia | *Anaerococcus* | 281 | SCZ only | Foxes < Skunks |
| * | Firmicutes | Clostridia | *Clostridium* | 257 | SCZ only | Foxes < Skunks |
| * | Firmicutes | Clostridia | *Dialister* | 281 | SCZ only | Foxes < Skunks |
| * | Firmicutes | Clostridia | *Peptoniphilus* | 280 | SCZ only | Foxes < Skunks |
| * | Actinobacteria | Actinobacteria | *Corynebacterium* | 251 | SCZ only | Foxes > Skunks |
| * | Bacteroidetes | Bacteroidia | *Butyricimonas* | 239 | SCZ only | Foxes > Skunks |
|  | Firmicutes | Clostridia | *-* | 259 | SCZ only | Foxes > Skunks |
|  | Firmicutes | Clostridia | *-* | 245 | SCZ only | Foxes > Skunks |
| * | Firmicutes | Clostridia | *Blautia* | 267 | SCZ only | Foxes > Skunks |
| * | Firmicutes | Clostridia | *Dorea* | 266 | SCZ only | Foxes > Skunks |
| * | Firmicutes | Clostridia | *Megamonas* | 246 | SCZ only | Foxes > Skunks |
| * | Firmicutes | Clostridia | *Oscillospira* | 248 | SCZ only | Foxes > Skunks |
| * | Firmicutes | Clostridia | *Phascolarctobacterium* | 272 | SCZ only | Foxes > Skunks |
| * | Proteobacteria | Epsilonproteobacteria | *Helicobacter* | 273 | SCZ only | Foxes > Skunks |
|  | Proteobacteria | Gammaproteobacteria | *-* | 264 | SCZ only | Foxes > Skunks |
|  | Proteobacteria | Gammaproteobacteria | *-* | 262 | SCZ only | Foxes > Skunks |
|  | Proteobacteria | Gammaproteobacteria | *-* | 260 | SCZ only | Foxes > Skunks |
| * | Actinobacteria | Actinobacteria | *Arcanobacterium* | 276 | SRI only | Foxes < Skunks |
| * | Actinobacteria | Actinobacteria | *Mobiluncus* | 282 | SRI only | Foxes < Skunks |
|  | Bacteroidetes | Bacteroidia | *-* | 273 | SRI only | Foxes < Skunks |
| * | Bacteroidetes | Bacteroidia | *Prevotella* | 271 | SRI only | Foxes < Skunks |
|  | Firmicutes | Bacilli | *-* | 272 | SRI only | Foxes < Skunks |
|  | Firmicutes | Clostridia | *-* | 276 | SRI only | Foxes < Skunks |
|  | Firmicutes | Clostridia | *-* | 276 | SRI only | Foxes < Skunks |
|  | Firmicutes | Clostridia | *-* | 266 | SRI only | Foxes < Skunks |
| * | Firmicutes | Clostridia | *Anaerococcus* | 282 | SRI only | Foxes < Skunks |
| * | Firmicutes | Clostridia | *Clostridium* | 233 | SRI only | Foxes < Skunks |
| * | Firmicutes | Clostridia | *Dialister* | 280 | SRI only | Foxes < Skunks |
|  | Firmicutes | Clostridia | *Epulopiscium* | 256 | SRI only | Foxes < Skunks |
|  | Firmicutes | Clostridia | *Gallicola* | 275 | SRI only | Foxes < Skunks |
|  | Firmicutes | Clostridia | *Helcococcus* | 255 | SRI only | Foxes < Skunks |
| * | Firmicutes | Clostridia | *Peptoniphilus* | 282 | SRI only | Foxes < Skunks |
|  | Fusobacteria | Fusobacteriia | *-* | 265 | SRI only | Foxes < Skunks |
|  | Proteobacteria | Epsilonproteobacteria | *-* | 258 | SRI only | Foxes < Skunks |
|  | Proteobacteria | Epsilonproteobacteria | *Campylobacter* | 270 | SRI only | Foxes < Skunks |
| * | Actinobacteria | Actinobacteria | *Corynebacterium* | 245 | SRI only | Foxes > Skunks |
|  | Actinobacteria | Coriobacteriia | *Collinsella* | 252 | SRI only | Foxes > Skunks |
|  | Bacteroidetes | Bacteroidia | *-* | 240 | SRI only | Foxes > Skunks |
|  | Bacteroidetes | Bacteroidia | *Alistipes* | 244 | SRI only | Foxes > Skunks |
|  | Bacteroidetes | Bacteroidia | *Bacteroides* | 241 | SRI only | Foxes > Skunks |
| * | Bacteroidetes | Bacteroidia | *Butyricimonas* | 238 | SRI only | Foxes > Skunks |
|  | Bacteroidetes | Bacteroidia | *Odoribacter* | 274 | SRI only | Foxes > Skunks |
|  | Bacteroidetes | Bacteroidia | *Parabacteroides* | 234 | SRI only | Foxes > Skunks |
|  | Bacteroidetes | Bacteroidia | *Prevotella* | 219 | SRI only | Foxes > Skunks |
|  | Firmicutes | Clostridia | *-* | 251 | SRI only | Foxes > Skunks |
|  | Firmicutes | Clostridia | *-* | 233 | SRI only | Foxes > Skunks |
|  | Firmicutes | Clostridia | *-* | 231 | SRI only | Foxes > Skunks |
|  | Firmicutes | Clostridia | *-* | 231 | SRI only | Foxes > Skunks |
| * | Firmicutes | Clostridia | *Blautia* | 280 | SRI only | Foxes > Skunks |
|  | Firmicutes | Clostridia | *Coprococcus* | 235 | SRI only | Foxes > Skunks |
| * | Firmicutes | Clostridia | *Dorea* | 245 | SRI only | Foxes > Skunks |
|  | Firmicutes | Clostridia | *Faecalibacterium* | 215 | SRI only | Foxes > Skunks |
| * | Firmicutes | Clostridia | *Megamonas* | 221 | SRI only | Foxes > Skunks |
| * | Firmicutes | Clostridia | *Oscillospira* | 252 | SRI only | Foxes > Skunks |
| * | Firmicutes | Clostridia | *Phascolarctobacterium* | 280 | SRI only | Foxes > Skunks |
|  | Firmicutes | Clostridia | *Ruminococcus* | 243 | SRI only | Foxes > Skunks |
|  | Fusobacteria | Fusobacteriia | *-* | 239 | SRI only | Foxes > Skunks |
|  | Proteobacteria | Alphaproteobacteria | *-* | 218 | SRI only | Foxes > Skunks |
|  | Proteobacteria | Deltaproteobacteria | *Bilophila* | 235 | SRI only | Foxes > Skunks |
|  | Proteobacteria | Deltaproteobacteria | *Desulfovibrio* | 258 | SRI only | Foxes > Skunks |
| * | Proteobacteria | Epsilonproteobacteria | *Helicobacter* | 258 | SRI only | Foxes > Skunks |
|  | Proteobacteria | Gammaproteobacteria | *-* | 264 | SRI only | Foxes > Skunks |
|  | Proteobacteria | Gammaproteobacteria | *-* | 247 | SRI only | Foxes > Skunks |
|  | Proteobacteria | Gammaproteobacteria | *-* | 233 | SRI only | Foxes > Skunks |
|  | Proteobacteria | Gammaproteobacteria | *Anaerobiospirillum* | 217 | SRI only | Foxes > Skunks |
|  | Actinobacteria | Coriobacteriia | *Collinsella* | 275 | Skunks only | SCZ > SRI |
|  | Fusobacteria | Fusobacteriia | *-* | 247 | Foxes only | SCZ > SRI |
